# On-Barn Forecasting Beef Cattle Production Based on Automated Non-Contact Body Measurement System

**DOI:** 10.3390/ani13040611

**Published:** 2023-02-09

**Authors:** Svetlana Gritsenko, Alexey Ruchay, Vladimir Kolpakov, Svyatoslav Lebedev, Hao Guo, Andrea Pezzuolo

**Affiliations:** 1 Agricultural Product Production and Processing Technology Department, South Ural State Agrarian University, 457100 Troitsk, Russia; 2 Federal Research Centre of Biological Systems and Agro-Technologies of the Russian Academy of Sciences, 460000 Orenburg, Russia; 3 Department of Mathematics, Chelyabinsk State University, 454001 Chelyabinsk, Russia; 4 Department of Biotechnology of Animal Raw Materials and Aquaculture, Orenburg State University, 460000 Orenburg, Russia; 5 College of Land Science and Technology, China Agricultural University, Beijing 100083, China; 6 Department of Land, Environment, Agriculture and Forestry, University of Padova, 35020 Legnaro, Italy; 7 Department of Agronomy, Food, Natural Resources, Animals and Environment, University of Padua, Padua, 35020 Legnaro, Italy

**Keywords:** precision livestock farming, beef cattle, non-contact body measurement, RGB-D camera, image analysis, machine learning

## Abstract

**Simple Summary:**

The aim of this study is to predict the productivity of beef cattle, using a systematic assessment of animals according to their main genetic parameters. Correlation analysis reveals that the main indices for the meat productivity prognosis are live weight and the measurements of animals taken at birth. Corresponding correlation coefficients were determined to predict animal body size at 18 months using measurements taken at birth. After our studies, it has been revealed that high positive correlation coefficients between individual traits (live weight and body measurements) indicate the expediency of using indirect selection. It has been established that three main indicators can serve as a forecast of meat productivity after slaughter in this population: the live weight of mothers, the live weight of animals at birth, and the indicators of animals at birth. The automated non-contact body measurement system using an RGB-D image capture system can be used to collect all three types of data that are necessary for accurate analysis.

**Abstract:**

The main task of selective breeding is to determine the early productivity of offspring. The sooner the economic value of an animal is determined, the more profitable the result will be, due to the proper estimation of high and low productive calves and distribution of the resources among them, accordingly. To predict productivity, we offer to use a systematic assessment of animals by using the main genetic parameters (correlation coefficients, heritability, and regression) based on data such as the measurement of morphological characteristics of animals, obtained using the automated non-contact body measurement system based on RGB-D image capture. The usefulness of the image capture system lies in significant time reduction that is spent on data collection and improvement in data collection accuracy due to the absence of subjective measurement errors. We used the RGB-D image capture system to measure the live weight of mother cows, as well as the live weight and body size of their calves (height at the withers, height in the sacrum, oblique length of the trunk, chest depth, chest girth, pastern girth). Cows and cattle of black-and-white and Holstein breeds (n = 561) were selected as the object of the study. Correlation analysis revealed the main indices for the forecast of meat productivity—live weight and measurements of animals at birth. Calculation of the selection effect is necessary for planning breeding work, since it can determine the value of economically beneficial traits in subsequent generations, which is very important for increasing the profitability of livestock production. This approach can be used in livestock farms for predicting the meat productivity of black-and-white cattle.

## 1. Introduction

Beef cattle production is estimated by livestock and post-slaughter indices. Required animal assessments (carried out by specialists) include body parameters such as live weight measurements of animals in different phases of production cycle, characterizing growth and development of the livestock in barn conditions. All of these data help to calculate the coefficients of heredity and the effect of selection, accordingly. 

Data on body weight and average daily gain of growing animals are the key factors for monitoring performance and for use in genetic evaluations for the purpose of achieving sustainable genetic gain. Accurate measurement of productivity, however, requires several measures of body weight over at least 70 days. This can be resource intensive, and thus, alternative approaches are required [1]. 

The development of precision livestock farming technologies and the constant growth of computing capabilities have created a new opportunity for effective solutions to the problems of assessing and predicting animal productivity. One of these solutions is to gather and use dynamic three-dimensional information about animals [2,3]. Modern digital methods of non-contact weight measurement can be carried out by a two-dimensional CCD camera [4,5] or three-dimensional camera [6]. The correlation coefficient is widely used in breeding work with cattle because it allows farm specialists and scientists to solve issues of planning and predicting the results of breeding [7,8]. For successful beef cattle production, one of the primary objectives is to define the economic value of an animal to lessen the cost of raising low-productive individuals, consequently increasing the profitability of the production [9,10,11].

The effect of selection is the main factor of breeding planning, which allows us to give an approximate forecast of what the productivity value of each animal will be in a year, or if there is a need for a generation change at the accepted level of selection under favorable and stable environmental conditions [1]. To account for this factor, it is necessary to determine the heritability coefficient of traits, which requires mass accounting and determination of indices of the animals’ own productivity and the productivity of their mothers (fathers) [12]. A genetic correlation has been established between the beef yield and the external sign of its musculature, which shows a strong relationship with net daily gain and body weight (0.79–0.97) [13]. However, the genetic correlations of cow carcass weight traits might be too large to ignore. Selection for steers with greater carcass weight would lead to larger cows [14].

Selection for beef traits in Italian dual-purpose breeds is often carried out using growth and in vivo conformation recorded on young, performance-tested bulls and muscularity traits scored during routinely linear-type evaluation on primiparous cows [15]. Along with this, image analysis methods can be considered as an improvement to the method of visual assessment of carcasses for the purpose of predicting the total yield of meat and variety in quality categories of commercial meat cuts from carcasses of young animals [16]. An approach to data analysis of dairy farms has been developed through the formulation of a properly integrated model for processing production and gathering behavioral data of every cow [17].

To predict productivity, we use a systematic assessment of animals by the main genetic parameters (correlation coefficients, heritability, and regression) based on data, including the measurement of morphological characteristics of animals obtained using the automated non-contact body measurement system based on RGB-D image capture. 

The main contribution of this work is as follows: (*i*) selection of the black-and-white and Holstein breeds cows and calves (n = 561); (*ii*) increasing the efficiency of breeding, based on high positive reliable correlation coefficients between individual traits (live weight and body measurements); (*iii*) the algorithm/approach was proposed for predicting the meat productivity of black-and-white bulls.

## 2. Materials and Methods

### 2.1. Animals and Ethics 

During the research, three experiments on three farms were conducted. Cows and bulls of black-and-white and Holstein breeds (n = 561) were selected as the object of the study. 

All methodologies and ethical standards were approved by the Committee for Control over the Maintenance and Use of Laboratory Animals in accordance with the “Policy of the Scientific Center for Work with Laboratory Animals” of the Federal State Budgetary Scientific Institution “Federal Research Center for Biological Systems and Agrotechnologies of the Russian Academy of Sciences”. An extract from the minutes of the meeting of the Commission for the Control of the Maintenance and Use of Laboratory Animals No. 4, dated 17 January 2021, was received on 20 March 2022. During the research, measures were taken to ensure the least suffering of animals and to reduce the number of experiment samples. 

The morphological characteristics of the animals were measured following the guidelines issued by the committee, and the data collection in this study was carried out without restriction of the animals. Because of that, performing this study does not require the approval of the Animal Care and Use Committee, which eliminates the need for the approval of environmental monitoring or animal science departments.

### 2.2. Tests and Data Collection

The first stage of research was to make the comparison between the dimensions and weight of each animal during its development. A total of 180 animals were kept on the JSC Breeding Plant “Russia” (Chelyabinsk, Russia), from birth to 18 months of age. An analysis of live weight and body measurements (height at the withers, height at the sacrum, oblique length of the trunk, chest depth, chest girth, pastern girth) at birth and the ages of 3, 6, 9, 12, 15 and 18 months were carried out. After calculating the correlation coefficients, we selected indices for predicting the meat productivity of animals.

**Figure 1:**
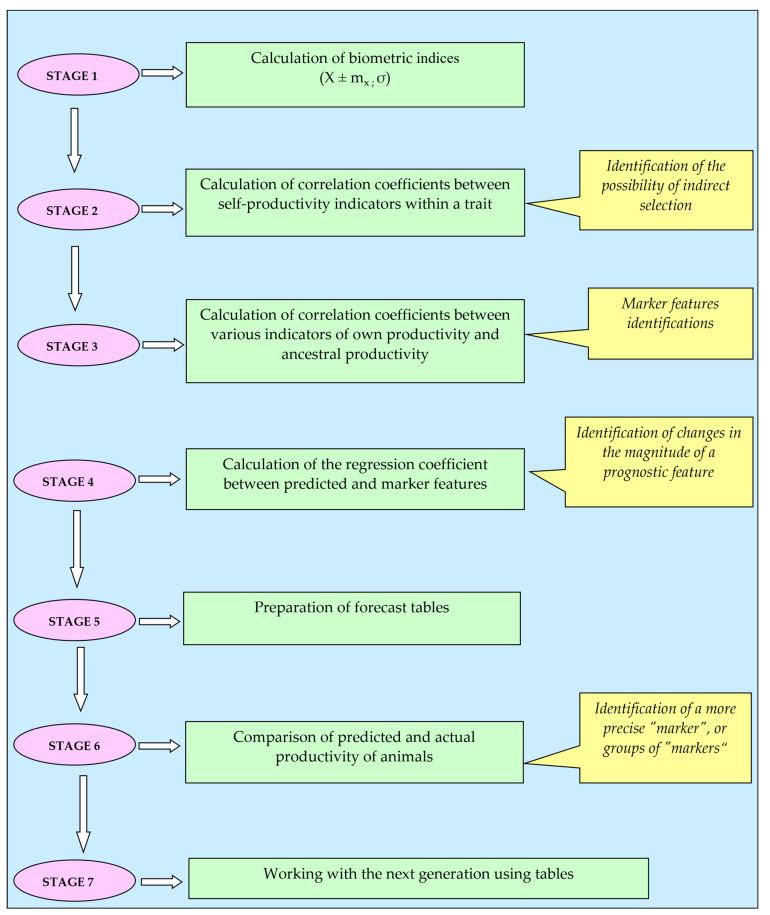
Productivity prediction algorithm.

In the second stage of research, the algorithm of applying genetic parameters to predict the meat productivity of animals (n = 201) was developed. To predict productivity, we used a systematic assessment of animals according to the main genetic parameters (correlation coefficients, heritability, and regression) (Figure 1). At the first stage, the forecast of the meat productivity of animals was made according to the indices, such as live weight of mothers, live weight, and height at the withers of animals at birth using forecast tables. In the second stage, the slaughter of the studied animals was carried out, and their actual post-slaughter indices were determined. At the end of the experiment, the animals were slaughtered at the commercial slaughterhouse owned by a meat processing plant. Animals were marked using an RFID chip. After the slaughter carcasses were marked using tags, they were refrigerated for 24–48 h. After refrigeration of the carcass, a deboning process was carried out. In the third research stage, the calculation was made to establish the accuracy of meat productivity forecast.

To predict productivity, a systematic assessment of animals according to the main genetic parameters (correlation coefficients, heritability, and regression) was advised, which consisted of the following stages:

1. Definition of animal groups and calculation of the main biometric indices of the studied traits (X ± mx; σ).
2. Calculation of correlation coefficients between indices of self-productivity within a body feature, to determine the directions and magnitude of the links between them, in order to identify the possibility of indirect selection, and finding the signs that suggest reduction of the productive body features due to negative communication directions.
3. Calculation of correlation coefficients between various indices of animal’s own productivity and the productivity of its ancestors, to identify signs markers for prognosis. These signs are interrelated with the studied productivity indices by reliably positive high correlation coefficients.
4. Calculation of the regression coefficient, to find the expected feature by increasing the marker feature by one. With regard of the regression coefficient, we used the previously calculated correlation coefficients of marker features and predicted indices, and the value of σ, which we calculated at the first stage of the forecast.
5. Using the average indices of the marker and predicted feature, the analysis was carried out in the first stage of the study, and the value of the regression coefficient was determined. Forecast tables were made.
6. The obtained results of the forecast tables were compared with the actual productivity of the studied animals to identify more accurate markers or groups of markers for a more precise prognosis result.
7. Used the appropriate forecast tables to work with the next generation of animals on this farm.

This approach aims to identify marker points for predicting the meat productivity of animals, mostly post-slaughter indices.

**Figure 2:**
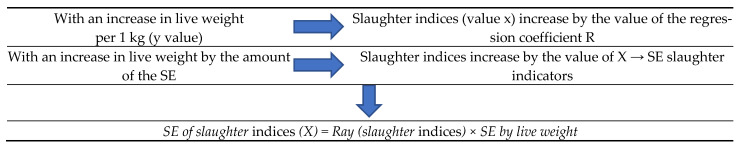
Calculation of the selection effect (SE) using the regression coefficient.

In the third stage of research, regression coefficients were used in assessing the effect of selection on meat productivity of black-and-white animals using the indices made after the slaughter. In total, 360 animals were analyzed (180 cattle and 180 cows, mothers of bulls of various origins). Research and calculation of meat productivity indices in cows and cattle were made during the livestock production (live weight, linear measurements), in addition to assessment of slaughter indices in animals (pre-slaughter mass, mass of paired carcass, mass of internal fat, slaughter mass, mass of chilled carcass, pulp mass, bone mass, tendon mass, half carcass mass). The selection effect was calculated using the regression coefficient (Figure 2).

### 2.3. RGB-D Image Capture System

**Figure 3:**
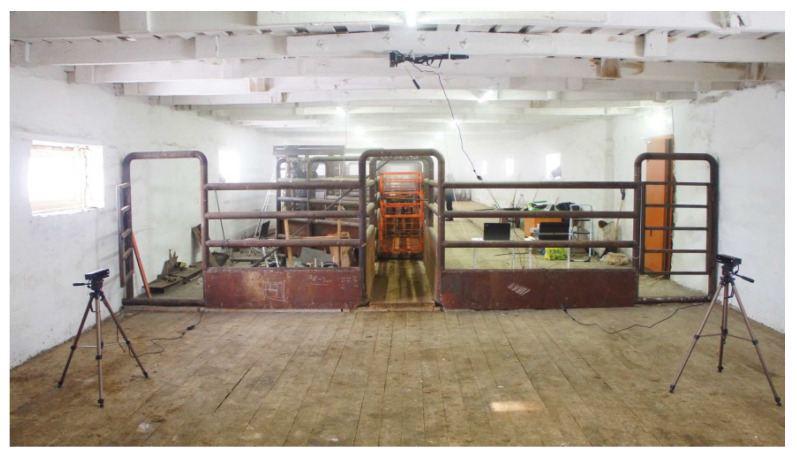
RGB-D image capture system with three Kinect cameras.

In each of the three research tests, an automated system for measuring the body parameters of live cattle was used [18]. The system is based on a non-rigid 3D shape reconstruction using data gathered from three depth cameras. The quality of measurements on generated 3D body models was compared with nine manually measured references. With a 90% confidence level, the system had less than 3% errors among all the measured estimates. Setup for data collection was placed in the passageway of the hall with the feeding system. All measurements were taken on a walking animal from three points of view since it was impossible to make the animals stop and remain motionless. The image capture system is shown in Figure 3. Two RGB-D cameras are located to the right- and left-sides of the animal passage with a distance approximately 2.0 m from the animal, and a third Kinect camera is located over the passage at approximately 3.0 m above the ground. The setup uses three identical Microsoft Kinect v2 cameras that acquire RGB and depth images from the left, right, and top views, capturing appropriate angles of the animal. Each camera is connected to a laptop via USB 3.0 cable. Synchronously captured RGB-D images from each camera were recorded to the corresponding laptop hard drive. 

Additionally, we used the machine learning model to predict the live weight of cattle [19]. This indirect automated estimation of the live weight of cattle consists of a non-invasive measurement of morphometric parameters of the livestock. The machine learning model uses both morphometric measurements and the age of the cattle. The accuracy of weight measurement using this model reaches 95.67%.

In the first experiment, we used the RGB-D image capture system to measure live weight and body size (height at the withers, height at the sacrum, oblique trunk length, chest depth, chest girth, pastern girth) at birth and at the ages of 3, 6, 9, 12, 15 and 18 months. In the second experiment, we used the RGB-D image capture system to measure the live weight of the calf’s mother, the live weight of the calf at birth and the height of the calf at the withers at birth.

## 3. Results

### 3.1. First Research Test

In the first experiment, we used the RGB-D image capture system to measure live weight and body size (height at the withers, height at the sacrum, oblique trunk length, chest depth, chest girth, pastern girth) at birth and at the ages of 3, 6, 9, 12, 15 and 18 months. Experimental results on real data showed that the RGB-D image capture system provides a high weight measurement accuracy of 92.4% and a high body measurement accuracy of 96.8%. 

The purpose of the first test was to use the calculation of correlation coefficients to find indices for predicting the meat productivity of cattle. It was found that sufficiently high reliable correlation coefficients were established between the live weight indices of animals in different periods of ontogenesis: live weight at birth and at 3, 6, 9, 12, 15 and 18 months in all the studied groups; indices ranged from 0.4 to 0.9. This indicates that the live weight of a calf at birth can be used to approximate its weight at 18 months of age and, in turn, making the earliest forecast of its further productivity (Table 1).

The correlation coefficients of the live weight of mothers are slightly lower (from 0.3 to 0.5). However, a positive relationship between live weight at birth and 18 months of age is maintained.

In all the studied groups, high positive, reliable correlation coefficients were determined between the body measurements of animals and live weight indices in different age periods. Because of this, the correlation coefficients between measurements at birth and live weight at 18 months of age ranged from 0.7 to 0.9, which indicates the possibility of using measurements of animals at birth to predict the pre-slaughter weight of the animal.

In support of this thesis, positive correlation coefficients were found between the measurements of mother cows and their live weight in different periods of ontogenesis. Positive associations with reliable highest indices of the correlation coefficient (g = 0.6–0.9) were confirmed between the measurements of the body of animals in all periods of ontogenesis, both in the general sample and in groups, depending on the linear origin and pedigree (Table 2).

This preliminary result indicates the potential of indirect selection by body measurements, which will increase the efficiency of selection according to the parameters of ontogenesis.

To identify the possibility of predicting body measurements at 18 months by measurements at birth, the corresponding correlation coefficients were decided. The established highly reliable relationship between these indices (g = 0.6–0.9) confirms this possibility.

### 3.2. Second Research Test

In the second experiment, we used the RGB-D image capture system to measure the live weight of the calf’s mother, the live weight of the calf at birth and the height of the calf at the withers at birth. Experimental results on real data showed that the RGB-D image capture system provides a high weight measurement accuracy of 91.7%. 

The main objective of this test is the research of marker factors to predict the meat productivity of cattle. It has been established that four main indices could serve as a forecast for meat productivity after slaughter in this population. Since correlation analysis has established a highly positive relationship between the studied parameters, each of them can serve as a marker for the forecast, especially the height at the withers of animals at birth. The connection between the slaughter indices helps to make a prognosis based on three main features—pre-slaughter mass, slaughter mass, and pulp mass.

**Figure 4:**
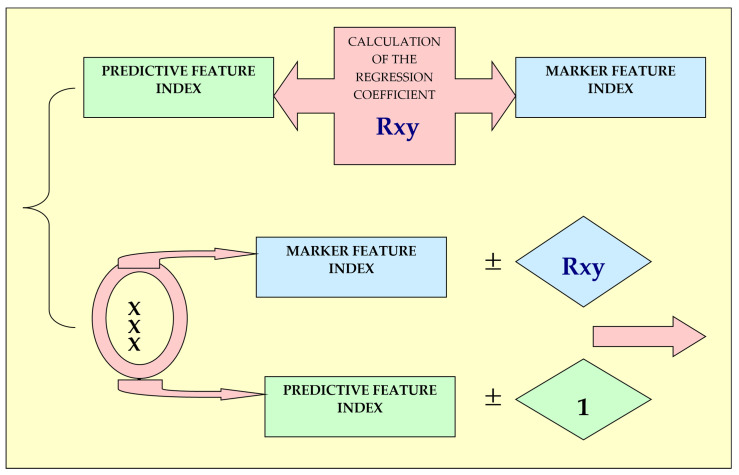
Diagram on the construction of the forecast table.

The next stage is the calculation of regression coefficients of predicted features through marker indices based on forecast tables (Table 3, Table 4 and Table 5). The diagram on the construction of the forecast table is shown in Figure 4.

It is established that the prediction of the main signs of meat productivity of an animal must be carried out by finding the arithmetic mean of forecast values of all the markers, for example, the live weight of the calf’s mother is 521 kg, live weight of a calf at birth is 27 kg, and the height at the withers of the calf at birth is 70 cm.

In Table 3, Table 4 and Table 5, each value of markers corresponds to the value of the forecast index and the calculation of arithmetic averages for it, and it establishes the estimated productivity of the bull (Table 6).

**Figure 5:**
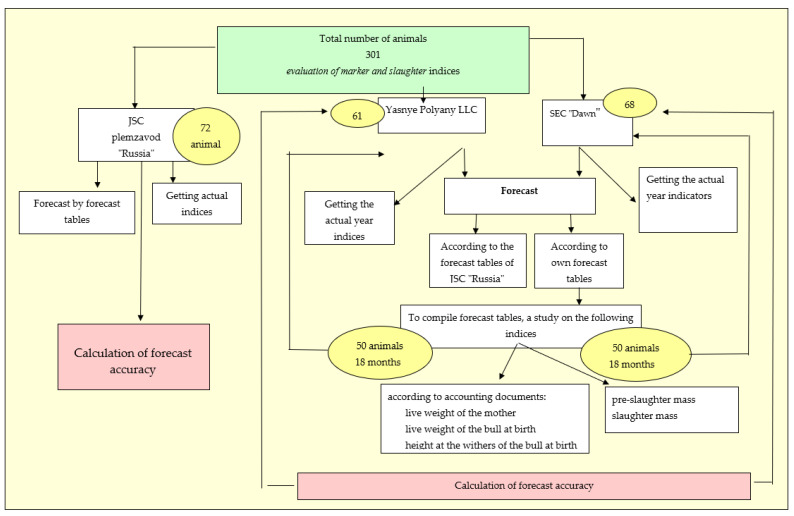
Scheme of approbation method of predicting meat productivity.

The approbation of this method for predicting the meat productivity of black-and-white cattle bulls was carried out on three farms: JSC “Russia”, LLC “Yasnye Polyany” and SEC “Dawn” (shown in diagram in Figure 5). On JSC breeding plant “Russia”, the research was carried out in three stages: at the first stage, the forecast of 72 animals’ meat productivity was made according to the indices of live weight of mothers, live weight and height at the withers of the calf at birth using forecast tables calculated based on this farm output (Table 3, Table 4 and Table 5); at the second stage, the slaughter of the studied animals was carried out, and their actual slaughter indices were determined; at the third stage, a calculation was made in order to assess the accuracy of prognosis.

On LLC “Yasnye Polyany” and SEC “Dawn”, same testing method was applied. The forecast of meat productivity of animals was carried out according to the forecast tables of JSC breeding plant “Russia” and accounting for its own forecast tables for three marker indices of 61 animals in LLC “Yasnye Polyany” and 68 animals of the SEC “Dawn”. To compile these forecast tables, we evaluated 50 heads of 18-month-old animals from each farm according to the relevant documentation of marker indices (live weight of the mother, live weight, and height at the withers of the bull at birth), slaughtering them afterward, with the assurance of the accuracy of meat productivity indices.

Furthermore, following all the stages of the forecast, appropriate algorithm calculations were carried out and forecast tables were compiled, resulting in expected indices of animal’s meat productivity being evaluated.

After 18 months, the slaughter of the studied animals was carried out, and actual slaughter was figured out, from which the accuracy of the forecast was calculated.

The approbation method of prognosis, carried out in a few farms in the Chelyabinsk region, has shown that the level of accuracy of the method ranges from 90 to 98% (Table 7).

The accuracy of the forecast increases by 3–6%, when the forecast tables for each farm are compiled using the proposed algorithm model.

### 3.3. Third Experiment 

The third experiment was executed on the JSC Breeding Plant “Russia”, with a total population of 360 animals (180 cattle and 180 cow mothers of bulls) of various origins. According to the indices of meat productivity assessed during the life of the animals, it was found that crossbreeds between the Vis Idial 933122 line and the Holstein breed produce animals with higher live weight values and bigger body dimensions in the next generation. 

The live weight of the offspring will increase by 0.9 kg at birth. On the pre-slaughter stage, weight of the next generation will be increased by 8.2 kg. The effect of breeding, regarding live weight, when using crossbreeds with ½ the blood of the Holstein breed, is somewhat lower since animals with ½ blood have a slightly reduced breeding differential. When using this breeding method, the live weight of descendants after a generation will increase by 0.6 kg at birth and at the pre-slaughter age by 7.4 kg.

Animal body dimensions increased by 1.0–2.0 cm in height at the withers, 2.3–2.1 cm in chest depth, and 2.6–2.4 cm in oblique trunk length.

The calculation of the breeding effect using only exterior indices does not provide a complete notion of breeding effectiveness on the meat productivity of animals. The most significant point in the assessment of selection effectiveness is the data characterization by post-slaughter indices; however, it is not possible to execute this by using the generally accepted method, because to calculate this, indices of the controlled slaughter of the studied mothers or fathers are required, which are not provided by the milk production technique. 

Regression coefficients show how much increases in value of the slaughtered cow will be if an increase in its live weight is by 1 kg. Knowing the regression coefficient of slaughter indices with a change in the live weight of animals by 1 kg, and the amount of live weight change in a year in a generation (selection effect), we can calculate the effectiveness of selection in slaughter indices via the effect of selection of the live weight index. To do this, we created a proportion (Figure 2).

It was found that in subsequent generations, the pre-slaughter weight of animals will increase by 10.2 and 6.8 kg (depending on the calculation by bloodlines); the mass of the paired carcass increases by 5.4 and 3.6 kg, the slaughter weight increase is by 5.3 and 3.6 kg, and the remaining indices will increase slightly from 0.1 to 2.0 kg.

## 4. Discussion

According to the data obtained by Guzzo et. al. [15], signs of linear musculature type were registered in 11,992 cows of the first pair, and the signs of this musculature type were evaluated in 957 young bull candidates. Heritability estimates obtained for muscularity traits were moderate in young bulls (on average 0.326), which is about 16% higher than in primiparous cows. Testing the average heritability for performance traits of young animals resulted in 0.342. Moderate to strong genetic correlations were found between performance test and muscularity type traits collected in young animals (varying from 0.500 between front (chest and shoulder) areas and average daily gain to 0.955 between thighs, buttocks side view and in vivo dressing percentage). The genetic relationships between muscularity linear type traits of primiparous cows and performance traits of young animals varied (from a null correlation between front (chest and shoulder) and average daily gain to 0.822 between thigh, buttock’s rear view and dressing percentage), with an average genetic correlation of 0.532. Generally, the traits measured in young animals during performance testing were favorably correlated with muscularity traits evaluated on primiparous cows, indicating a matching selection pattern [15].

Accurate estimates of genetic merit for both live weight and body condition score could be useful additions to both national- and herd-breeding programs [20]. Although recording live weight and body condition score is not technologically challenging, available data for use in routine genetic evaluations are generally lacking [21]. Overall, linear type trait data are a useful source of information for the purpose of predicting live weight and body condition score with minimal additional predictive values, while also including carcass data. Nonetheless, with the absence of linear type trait data, most of the information on carcass traits can be used to predict genetic merit for mature cow live weight [22,23,24].

In our study, positive associations with high and reliable indices of the correlation coefficient (g = 0.6–0.9) were validated during the measurements of the body of animals in all periods of ontogenesis, both in the general sample and in groups, depending on the linear origin and pedigree.

According to other data, estimates of genetic correlations ranged from 0.23 to 0.94 among growth traits, indicating that selection based on these traits can be successful in breeding programs [25]. On the other hand, according to other results, heritability estimates can range from 0.38 (36 months) to 0.78 (94 months) with fluctuations, especially for extreme ages. Estimates of genetic correlations were high for most age pairs, with the lowest score (0.70) between the extreme ages (19 and 103 months) [26]. This, in our opinion, indicates the expediency of using an indirect selection by body measurements, which will increase the efficiency of selection by the parameters of ontogenesis.

Since the correlation analysis has established a high positive relationship between the studied parameters, each of them can serve as a marker for the forecast.

The method and the model used for the genetic evaluation of specific traits in a certain breeding organization are important for: the exact definition of traits, determination of the economic values of each animal, and the inclusion or exclusion of links between traits in the calculation of the economic values of livestock breeding [27].

Genomic selection improves the possibility of using breeding techniques in the conditions of a specified farm [28]. As an example, knowing the regression coefficient of slaughter indices when the live weight of animals changes by 1 kg, and the amount of change in live weight of generation in a year (selection effect), we can calculate the effectiveness of selection in terms of slaughter indices, using the live weight selection effect index. It was found that in subsequent generations, the pre-slaughter weight of cattle will increase by 10.2 and 6.8 kg (depending on the calculation by bloodline); the mass of the paired carcass increases by 5.4 and 3.6 kg, the slaughter weight increases by 5.3 and 3.6 kg, and the remaining indices will slightly increase from 0.1 to 2.0 kg.

Even though the pace of change and the direction of development of animal husbandry have immense variety in different regions of the world, no significant changes in beef production methods can be expected until 2050 [29,30].

## 5. Conclusions

The correlation analysis revealed that the main marker indices for the forecast of meat productivity are live weight and dimensions of animals at birth. In addition, high positive reliable correlation coefficients have been validated between individual traits (live weight, animal dimensions), which allows for the use of indirect selection to increase the efficiency of breeding.

We proposed the algorithm and the method for predicting the meat productivity of black-and-white bulls that can be used as a practical solution for agricultural enterprises. The approbation of this forecasting method, used in a few farms in the Chelyabinsk region, has revealed that the accuracy level of this method ranges from 90 to 98%.

Calculation of the selection effect is necessary for breeding planning, as it helps to determine the value of economically beneficial traits in subsequent generations, which is a very important factor in increasing the profitability of animal husbandry. 

In the future, we plan to test this method of predicting productivity on the specialized beef cattle livestock, to analyze and compare the results with the data obtained in this research.

## Figures and Tables

**Table 1:** Correlation coefficients between indices of live weight of bulls in different periods of ontogenesis.

Sign		Bull Line			Bloodline			Total by Group
		Franc 10736366	Vis Idiala 933122	Siling Trijun 252803	Black and White	*^1/2^* Holstein	*^1/4^* Holstein	
At birth	3 months	0.8 ± 0.06 *	0.9 ± 0.03 *	0.9 ± 0.03 *	0.9 ± 0.03 *	0.8 ± 0.06 *	0.8 ± 0.06 *	0.9 ± 0.04 *
	6 months	0.4 ± 0.2 *	0.5 ± 0.1 *	0.5 ± 0.1 *	0.6 ± 0.1 *	0.5 ± 0.1 *	0.4 ± 0.2 *	0.5 ± 0.08 *
	9 months	0.5 ± 0.1 *	0.6 ± 0.01 *	0.6 ± 0.1 *	0.5 ± 0.1 *	0.6 ± 0.1 *	0.6 ± 0.1 *	0.6 ± 0.07 *
	12 months	0.7 ± 0.09 *	0.7 ± 0.09 *	0.7 ± 0.09 *	0.7 ± 0.09 *	0.7 ± 0.09 *	0.7 ± 0.09 *	0.9 ± 0.02 *
	15 months	0.8 ± 0.06 *	0.8 ± 0.06 *	0.7 ± 0.09 *	0.8 ± 0.06 *	0.8 ± 0.06 *	0.8 ± 0.06 *	0.9 ± 0.02 *
	18 months	0.8 ± 0.06 *	0.9 ± 0.03 *	0.8 ± 0.06 *	0.7 ± 0.09 *	0.9 ± 0.03 *	0.7 ± 0.09 *	0.9 ± 0.02 *
3 months	6 months	0.4 ± 0.2 *	0.5 ± 0.1 *	0.4 ± 0.2 *	0.5 ± 0.1 *	0.5 ± 0.1 *	0.5 ± 0.09 *	0.5 ± 0.08 *
	9 months	0.6 ± 0.1 *	0.6 ± 0.1 *	0.6 ± 0.1 *	0.7 ± 0.09 *	0.6 ± 0.1 *	0.5 ± 0.1 *	0.6 ± 0.07 *
	12 months	0.9 ± 0.03 *	0.8 ± 0.06 *	0.8 ± 0.06 *	0.9 ± 0.03 *	0.8 ± 0.06 *	0.8 ± 0.06 *	0.9 ± 0.02 *
	15 months	0.8 ± 0.06 *	0.8 ± 0.06 *	0.9 ± 0.03 *	0.8 ± 0.06 *	0.9 ± 0.03 *	0.9 ± 0.03 *	0.9 ± 0.02 *
	18 months	0.7 ± 0.09 *	0.6 ± 0.1 *	0.7 ± 0.09 *	0.8 ± 0.06 *	0.9 ± 0.03 *	0.8 ± 0.06 *	0.9 ± 0.02 *
6 months	9 months	0.4 ± 0.2 *	0.5 ± 0.1 *	0.5 ± 0.1 *	0.7 ± 0.09 *	0.7 ± 0.09 *	0.7 ± 0.09 *	0.7 ± 0.05 *
	12 months	0.5 ± 0.1 *	0.4 ± 0.2 *	0.5 ± 0.1 *	0.6 ± 0.1 *	0.5 ± 0.1 *	0.5 ± 0.3 *	0.5 ± 0.08 *
	15 months	0.4 ± 0.2 *	0.5 ± 0.1 *	0.6 ± 0.1 *	0.5 ± 0.1 *	0.5 ± 0.1 *	0.5 ± 0.2 *	0.5 ± 0.08 *
	18 months	0.5 ± 0.1 *	0.5 ± 0.1 *	0.5 ± 0.1 *	0.5 ± 0.1 *	0.4 ± 0.2 *	0.5 ± 0.03 *	0.5 ± 0.08 *
9 months	12 months	0.6 ± 0.1 *	0.7 ± 0.09 *	0.7 ± 0.09 *	0.6 ± 0.1 *	0.7 ± 0.09 *	0.6 ± 0.1 *	0.6 ± 0.07 *
	15 months	0.5 ± 0.1 *	0.5 ± 0.1 *	0.4 ± 0.2 *	0.6 ± 0.1 *	0.5 ± 0.1 *	0.6 ± 0.08 *	0.6 ± 0.07 *
	18 months	0.5 ± 0.1 *	0.5 ± 0.1 *	0.5 ± 0.1 *	0.6 ± 0.1 *	0.6 ± 0.1 *	0.6 ± 0.1 *	0.6 ± 0.07 *
12 months	15 months	0.8 ± 0.06 *	0.8 ± 0.06 *	0.9 ± 0.03 *	0.8 ± 0.06 *	0.8 ± 0.06 *	0.9 ± 0.03 *	0.9 ± 0.02 *
	18 months	0.8 ± 0.06 *	0.7 ± 0.09 *	0.7 ± 0.09 *	0.9 ± 0.03 *	0.8 ± 0.06 *	0.9 ± 0.03 *	0.9 ± 0.02 *
15 months	18 months	0.7 ± 0.09 *	0.9 ± 0.03 *	0.8 ± 0.06 *	0.9 ± 0.03 *	0.8 ± 0.06 *	0.9 ± 0.03 *	0.9 ± 0.02 *

* here and further reliable correlation coefficients.

**Table 2:** Correlation coefficients between the indices of bullhead measurements in different age periods.

Correlated Features	Height at the Withers	Height in the Sacrum	Oblique Length of the Trunk	Chest Depth	Chest Width	Width in Makloks	Chest Girth	Butt Half—Girth
At birth								
Height in the sacrum	0.9 *	-	-	-	-	-	-	-
Oblique length of the trunk	0.9 *	0.9 *	-	-	-	-	-	-
Chest depth	0.9 *	0.9 *	0.8 *	-	-	-	-	-
Chest width	0.9 *	0.9 *	0.8 *	0.9 *	-	-	-	-
Width in makloks	0.9 *	0.9 *	0.8 *	0.9 *	0.9 *	-	-	-
Chest girth	0.9 *	0.9 *	0.9 *	0.9 *	0.9 *	0.9 *	-	-
Butt half-girth	0.9 *	0.9 *	0.9 *	0.9 *	0.9 *	0.9 *	0.9 *	-
Pastern girth	0.9 *	0.7 *	0.8 *	0.9 *	0.9 *	0.9 *	0.9 *	0.9 *
At 3 months								
Height in the sacrum	0.9 *	-	-	-	-	-	-	-
Oblique length of the trunk	0.9 *	0.9 *	-	-	.	-	-	-
Chest depth	0.9 *	0.9 *	0.8 *	-	-	-	-	-
Chest width	0.9 *	0.9 *	0.8 *	0.9 *	-	-	-	-
Width in makloks	0.9 *	0.9 *	0.8 *	0.9 *	0.9 *	-	-	-
Chest girth	0.9 *	0.9 *	0.9 *	0.9 *	0.9 *	0.9 *	-	-
Butt half-girth	0.9 *	0.9 *	0.9 *	0.9 *	0.9 *	0.9 *	0.9 *	-
Pastern girth	0.9 *	0.9 *	0.8 *	0.9 *	0.9 *	0.9 *	0.9 *	0.9 *
At 6 months								
Height in the sacrum	0.9 *	-	-	-	-	-	-	-
Oblique length of the trunk	0.9 *	0.9 *	-	-	-	-	-	-
Chest depth	0.9 *	0.9 *	0.9 *	-	-	-	-	-
Chest width	0.8 *	0.9 *	0.9 *	0.8 *	-	-	-	-
Width in makloks	0.9 *	0.7 *	0.8 *	0.8 *	0.8 *	-	-	-
Chest girth	0.9 *	0.9 *	0.9 *	0.9 *	0.9 *	0.8 *	-	-
Butt half-girth	0.9 *	0.9 *	0.9 *	0.9 *	0.9 *	0.8 *	0.9 *	-
Pastern girth	0.3 *	0.7 *	0.8 *	0.7 *	0.6 *	0.7 *	0.8 *	0.8 *
At 9 months								
Height in the sacrum	0.8 *	-	-	-	-	-	-	-
Oblique length of the trunk	0.9 *	0.6 *	-	-	-	-	-	-
Chest depth	0.8 *	0.7 *	0.8 *	-	-	-	-	-
Chest width	0.8 *	0.6 *	0.8 *	0.7 *	-	-	-	-
Width in makloks	0.9 *	0.7 *	0.9 *	0.8 *	0.8 *	-	-	-
Chest girth	0.9 *	0.8 *	0.9 *	0.8 *	0.8 *	0.8 *	-	-
Butt half-girth	0.9 *	0.9 *	0.9 *	0.9 *	0.7 *	0.8 *	0.9 *	-
Pastern girth	0.8 *	0.7 *	0.8 *	0.7 *	0.6 *	0.7 *	0.8 *	0.8 *
At 15 months								
Height in the sacrum	0.9 *	-	-	-	-	-	-	-
Oblique length of the trunk	0.9 *	0.9 *		-	-	-	-	-
Chest depth	0.9 *	0.9 *	0.9 *	-	-	-	-	-
Chest width	0.9 *	0.9 *	0.9 *	0.9 *	-	-	-	-
Width in makloks	0.9 *	0.9 *	0.9 *	0.9 *	0.9 *	-	-	-
Chest girth	0.9 *	0.9 *	0.9 *	0.9 *	0.9 *	0.9 *	-	-
Butt half-girth	0.9 *	0.9 *	0.9 *	0.9 *	0.9 *	0.9 *	0.9 *	-
Pastern girth	0.9 *	0.9 *	0.9 *	0.9*	0.9 *	0.9 *	0.9 *	0.9 *
At 18 months								
Height in the sacrum	0.9 *	-	-	-	-	-	-	-
Oblique length of the trunk	0.9 *	0.9 *	-	-	. -	-	-	-
Chest depth	0.9 *	0.9 *	0.9 *	-	-	-	-	-
Chest width	0.9 *	0.9 *	0.9 *	0.9 *	-	-	-	-
Width in makloks	0.9 *	0.9 *	0.9 *	0.9 *	0.9 *	-	-	-
Chest girth	0.9 *	0.9 *	0.9 *	0.9 *	0.9 *	0.9 *	-	-
Butt half-girth	0.9 *	0.9 *	0.9 *	0.9 *	0.9 *	0.9 *	0.9 *	-
Pastern girth	0.9 *	0.9 *	0.9 *	0.9 *	0.9 *	0.9 *	0.9 *	0.9 *

* here and further reliable correlation coefficients.

**Table 3:** Table for predicting the meat productivity of animals by the live weight of mothers.

Index	Regression Coefficient	Live Weight of Mothers						Average Indices		Live Weight of Mothers					
		506	507	508	509	510	511	Live Weight	Slaughter Weight	513	514	515	516	517	521
Pre-slaughter weight, kg	1.3	417.7	419.0	420.4	421.7	423.0	424.4	512	425.7	427.0	428.4	429.7	431.0	432.4	437.7
Slaughter weight, kg	0.8	232.4	233.2	234.0	234.8	235.7	236.5	512	237.3	238.1	238.9	239.8	240.6	241.4	244.7
Pulp weight, kg	0.6	171.9	172.4	172.9	173.5	174.1	174.6	512	175.2	175.8	176.3	176.8	177.4	177.9	180.2

**Table 4:** Table for predicting the meat productivity of animals by their live weight at birth.

Index	Regression Coefficient	Live Weight of a Calf at Birth						Average Indices		Live Weight of a Calf at Birth					
		20	21	22	23	24	25	Live Weight	Slaughter Weight	27	28	29	30	31	32
Pre-slaughter weight, kg	11.3	357.7	369.03	380.4	391.7	403.0	414.4	26	425.7	437.0	448.4	459.7	471.0	482.4	493.7
Slaughter weight, kg	6.0	201.3	207.3	213.3	219.3	225.3	231.3	26	237.3	243.3	249.3	255.3	261.3	267.3	273.3
Pulp weight, kg	4.7	147.0	151.7	156.4	161.1	165.8	170.5	26	175.2	179.9	184.6	189.3	194.0	198.7	203.4

**Table 5:** Table for predicting the meat productivity of animals by their height at the withers at birth.

Index	Regression Coefficient	Height at the Withers of the Calf at Birth						Average Indices		Height at the Withers of the Calf at Birth					
		64	65	66	67	68	69	Height at the Withers	Slaughter Weight	71	72	73	74	75	76
Pre-slaughter weight, kg	5.1	395.1	400.2	405.3	410.4	415.5	420.6	70	425.7	430.8	435.9	441.0	446.1	451.2	456.3
Slaughter weight, kg	2.8	220.5	223.3	226.1	228.9	231.7	234.5	70	237.3	240.1	242.9	245.7	248.5	251.3	254.1
Pulp weight, kg	1.9	163.9	165.8	167.7	169.6	171.4	173.3	70	175.2	177.1	178.9	180.8	182.7	184.6	186.5

**Table 6:** Calculation of forecast indices for meat productivity.

Meat Productivity Index	Marker Indices			Estimated Productivity (Arithmetic Mean by Markers)
	Live Weight of Mothers	Live Weight of a Calf at Birth	Height at the Withers of the Calf at Birth	
	521	27	70	-
Pre-slaughter weight, kg	437.7	437.0	425.7	433.5
Slaughter weight, kg	244.7	243.3	237.3	241.7
Pulp weight, kg	180.2	179.9	175.2	178.4

**Table 7:** Results of approbation of the method of forecasting the meat productivity of animals in the farms of the Chelyabinsk region.

Farm	Index (kg)	Animals	Forecast According to Forecast Tables of JSC “Russia”			Forecast According to Forecast Tables Compiled in Farms			
			Productivity, kg		Forecast Accuracy, %	Productivity, kg		Forecast Accuracy, %	
			Forecast	Actual		Forecast	Actual		
JSC “Russia”	Pre-slaughter weight	72	428.6 ± 1.7	420.3 ± 1.8	98	-		--	
	Slaughter weight		236.3 ± 1.3	229.2 ± 1.2	97	-	-	-	
Yasnye Polyany LLC	Pre-slaughter weight	61	419.3 ± 2.6	382.4 ± 2.5	91	393.0 ± 2.7	382.4 ± 2.5	97	
	Slaughter weight		234.1 ± 1.5	210.3 ± 1.4	90	218.1 ± 1.4	210.3 ± 1.4	96	
SEC “Dawn”	Pre-slaughter weight	68	423.2 ± 2.5	398.8 ± 2.5	94	410.9 ± 2.5	398.8 ± 2.5	97	
	Slaughter weight		232.6 ± 1.4	218.1 ± 1.2	93	222.4 ± 1.3	218.1 ± 1.2	98	

## Data Availability

The data presented in this study are available.
